# Brainstem ^1^H-MR spectroscopy in episodic and chronic migraine

**DOI:** 10.1007/s10194-012-0491-0

**Published:** 2012-10-16

**Authors:** Tzu-Hsien Lai, Jong-Ling Fuh, Jiing-Feng Lirng, Ching-Po Lin, Shuu-Jiun Wang

**Affiliations:** 1 School of Medicine, National Yang-Ming University, Taipei, Taiwan; 2 Institute of Neuroscience, National Yang-Ming University, Taipei, Taiwan; 3 Division of Neurology, Department of Internal Medicine, Far Eastern Memorial Hospital, New Taipei, Taiwan; 4 Department of Neurology, Neurological Institute, Taipei Veterans General Hospital, No. 201, Sec. 2, Shipai Rd, Beitou District, Taipei, 11217 Taiwan; 5 Department of Radiology, Taipei Veterans General Hospital, Taipei, Taiwan

**Keywords:** Brainstem, Periaqueductal gray, Pons, Migraine, MR spectroscopy

## Abstract

The pathogenesis of evolution from episodic migraine (EM) to chronic migraine (CM) has not yet been clearly determined. Some studies revealed that dysfunction of the brainstem may play a role. We aimed to determine the brainstem ^1^H-MR spectroscopic (MRS) findings in episodic and chronic migraine. We recruited patients with EM, CM and controls. Patients with CM were divided into subgroups with and without medication overuse (MO). The ^1^H-MRS metabolite ratios at the periaqueductal gray (PAG) and bilateral dorsal pons were measured and compared with those in controls. A total of 19 patients with EM, 53 patients with CM (with MO *n* = 30, without MO *n* = 23) and 16 control subjects completed the study. Patients with EM had the highest *N*-acetylaspartate (NAA)/creatine (Cr) ratio at the dorsal pons (right, *P* = 0.014; left, *P* = 0.034) in comparison with those of CM and controls. The latter two groups did not differ. Among migraine patients, NAA/Cr ratios at dorsal pons were inversely correlated with headache frequency (right, *r* = −0.350, *P* = 0.004; left, *r* = −0.284, *P* = 0.019) and intensity (right, *r* = −0.286, *P* = 0.019; left, *r* = −0.244, *P* = 0.045), but not disease duration. In contrast, the metabolite ratios did not differ at the PAG among the study groups. Of note, MO was not associated with brainstem MRS ratios in patients with CM. The increased NAA/Cr levels may suggest neuronal hypertrophy at the dorsal pons in EM. A progressive dysfunction of this region may occur from EM to CM since the levels declined with increasing headache frequency and intensity.

## Introduction

Migraine is traditionally recognized as a purely episodic disorder that patients are left without sequela [1]. A longitudinal population-based study has shown that about 2.5 % of patients with episodic migraine (EM) progress to chronic migraine (CM) annually [2], defined as headache attacks ≥15 days/month with ≥8 days of migraine attacks, for more than 3 months [3]. CM is associated with more severe disability, higher medical costs, lower quality of life, higher percentages of psychiatric comorbidities, and possibly a higher risk of brain damage [4–7]. Though many risk factors such as obesity and medication overuse (MO) have been identified, the mechanism of disease evolution is still unknown [8].

Brainstem has been suggested as “the generator” of migraine attacks [9–14]. Functional neuroimaging studies have shown the activation of the brainstem during migraine attacks, either spontaneous or triggered [9–12]. Structural changes at the periaqueductal gray (PAG) and dorsal pons have also been reported in patients with migraine [13, 14]. In contrast, the involvement of brainstem may not be specific for migraine, since activation and structural changes are also identified in other pain disorders [15, 16].

We conducted a study of proton MR spectroscopy (^1^H-MRS) over the PAG and dorsal pontine regions in patients with EM or CM (with or without MO). ^1^H-MRS can provide regional metabolite levels including *N*-acetylaspartate (NAA), choline containing phospholipids (Cho), creatine and phosphocreatine (Cr), etc. that can be used to investigate the pathophysiology of neurological disorders [17, 18]. NAA/Cr is generally considered to be a marker of neurons, being reduced in conditions where there is neuronal loss or dysfunction [19, 20]. Serum NAA level has been reported lower in patients with migraine, compared with that in patients with tension-type headache and healthy controls [21]. Cho/Cr can be viewed as an indirect marker of myelination and cell membrane metabolism [19, 20]. By recruiting both patients with EM and CM, we aimed to investigate the metabolite changes in the brainstem, which might shed light on the mechanism of migraine evolution. In addition, we also tested if there was any relationship between brainstem and MO, a known risk factor of migraine evolution.

## Methods

### Subjects

We recruited patients from the Headache Clinic at Taipei Veterans General Hospital (VGH). The diagnoses of EM and CM (with or without MO) were made according to the International Classification of Headache Disorders, 2nd edition (code 1.1 and A1.5.1) (ICHD-2) [3, 22]. MO was defined as ≥10 or 15 days of using abortive agents per month for more than 3 months [22]. Demographics, headache characteristics, body mass index (BMI), Beck Depression Inventory (BDI) and Migraine Disability Assessment Scale (MIDAS) were recorded in a structured questionnaire before the MRS study. The monthly headache frequency and pain intensity (0–10 numerical rating scale) were recorded. Age and sex-similar healthy subjects were recruited from the colleagues of Taipei-VGH as controls, who had no headache or had infrequent tension-type headache, i.e. <1 day per month [22]. The study protocol was approved by the Institutional Review Board of Taipei-VGH. Each subject provided written informed consent before entering the study.

### Multi-voxel ^1^H-MRS of PAG and bilateral dorsal pons

**Fig. 1:**
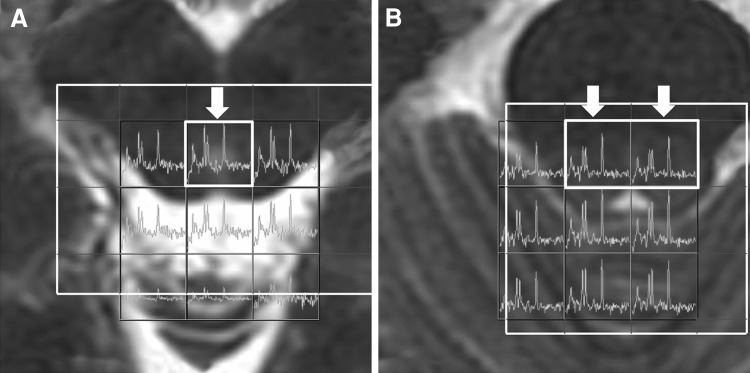
The voxels of interest of MR spectroscopic study were located at the periaqueductal gray matter (**a**) in midbrain and bilateral rostral dorsal pons (**b**)

All intracranial two-dimensional multi-voxel MRS studies using point-resolved spectroscopy (PRESS) localization (TR 1,000 ms, TE 144 ms, FOV 24 cm, 18 × 18 phase encoding matrices, 1.0 cm section thickness) with automated shim and water suppression (PROBE-P, Version 8.3, GE Medical Systems, Milwaukee, WI, USA) were acquired on a 1.5 T GE Signa Excite scanner. The localized voxels of interest (VOIs) were placed over the PAG and bilateral dorsal aspects of the rostral pons (Fig. 1) by the same neuroradiologist (Lirng JF). The MRS scan was initiated if the line width reported by the prescan process was less than 6 Hz. Each study lasted up to 7 min including the prescan process. Off-line spectral post-processing was carried out using semi-automated software (Probe 2000, Functool, Version 2.33, GE Medical Systems, Milwaukee, WI, USA). Spectra were displayed as grids of nominal voxel size 7.5 mm × 7.5 mm × 10 mm and overlaid on the conventional MR image used to plan the study. Spectral peaks of the main metabolites–Cho at 3.2 ppm, Cr at 3.0 ppm and NAA at 2.0 ppm were analyzed and the ratios between the metabolites (Cho/Cr and NAA/Cr) were calculated by Functool automatically.

### Statistical analysis

All the statistics were done with the software of SPSS for Windows version 18.0 (SPSS Inc., Chicago). For continuous data comparison among three groups (control, EM and CM), one-way analysis of variance (ANOVA) followed by post hoc analysis with least significant difference (LSD) method was used. For continuous data comparison between two groups, independent sample *t* test was used. For categorical data, Chi-square test or Fisher’s exact test was used. Pearson correlation was performed to identify possible associations between continuous variables. General linear model (GLM) was used to control confounding factors. All tests were two-tailed and *P* < 0.05 was considered significant.

## Results

### Demographics

A total of 72 patients with migraine [EM = 19, CM = 53 (with MO = 30, without MO = 23)] and 16 healthy controls were recruited for the study. All patients and controls were right handed. The demographics and headache profiles are listed in Table 1. These three groups were rather compatible except that patients with CM, compared to patients with EM, had longer disease duration, more days of headache and usage of analgesics per month, and a higher disability as assessed by MIDAS. Patients with CM had a nonsignificantly higher BDI score than those with EM (*P* = 0.075).

**Table 1:** Comparisons of demographics and headache profiles between episodic and chronic migraine patients and normal controls

	C (*n* = 16)	EM (*n* = 19)	CM (*n* = 53)	*P*
Age, years	39.6 ± 8.3	35.2 ± 10.5	40.6 ± 10.5	0.133
Female, *n* (%)	10 (62.5)	12 (63.2)	44 (83.0)	0.102
Age at onset, years	NA	19.4 ± 8.1	21.4 ± 8.4	0.425
Disease duration, years	NA	14.3 ± 7.6	19.7 ± 10.1	0.049*
Headache intensity^a^	NA	6.7 ± 2.0	7.1 ± 2.3	0.566
Headache frequency, days/month	NA	7.5 ± 3.5	24.7 ± 5.8	<0.001*
Analgesics use, days/month	NA	4.1 ± 4.5	16.3 ± 11.7	<0.001*
MIDAS (0–270)	NA	19.6 ± 19.5	62.8 ± 56.1	0.005*
BDI score (0–63)	NA	9.9 ± 7.5	14.6 ± 9.2	0.075
BMI (kg/m^2^)	NA	22.4 ± 3.4	22.5 ± 5.3	0.930
Unilateral, *n* (%)	NA	12 (66.7)	27 (51.9)	0.278
Throbbing headache, *n* (%)	NA	12 (66.7)	38 (73.1)	0.604
Physical activity aggravation, *n* (%)	NA	15 (83.3)	40 (76.9)	0.568
Associated symptoms				
Nausea, *n* (%)	NA	12 (66.7)	37 (71.2)	0.720
Vomiting, *n* (%)	NA	7 (38.9)	26 (50.0)	0.416
Photophobia, *n* (%)	NA	10 (58.8)	35 (67.3)	0.566
Phonophobia, *n* (%)	NA	13 (72.2)	39 (75.0)	1.0

*C* control, *EM* episodic migraine, *CM* chronic migraine, *NA* not applicable, *MIDAS* migraine disability assessment scale, *BDI* Beck depression inventory, *BMI* body mass index

* *P* < 0.05

^a^Headache intensity in a 0–10 numerical rating scale

### ^1^H-MRS differences among different groups

The results of MRS in the PAG and bilateral dorsal pons are shown in Table 2. The difference was not significant for PAG among the three study groups. Regarding dorsal pons, one outliner data of right dorsal pons was found in the EM group (NAA/Cr = 4.73, mean/standard deviation (SD) = 2.02/0.74, *z* = 3.67; Cho/Cr = 4.75, mean/SD = 1.61/0.82, *z* = 3.83). The results in Table 2 and the following calculations were done after removal of this outlier data. NAA/Cr ratio was higher in patients with EM at the dorsal pons (right side, *P* = 0.014; left side, *P* = 0.034) in comparison with the other two groups. Post hoc analysis revealed significantly higher ratios in EM patients as compared with CM patients (right side, *P* = 0.004; left side, *P* = 0.017) and controls (right side, *P* = 0.033; left side, *P* = 0.025); whereas, no difference was found between patients with CM and controls (right side, *P* = 0.829; left side, *P* = 0.655). Among all migraine patients (EM + CM), significant inverse correlations were found between dorsal pons NAA/Cr ratios and headache frequency (right side, *r* = −0.350, *P* = 0.004; left side, *r* = −0.284, *P* = 0.019) and headache intensity (right side, *r* = −0.286, *P* = 0.019; left side, *r* = −0.244, *P* = 0.045). No correlations were noted between NAA/Cr ratios in dorsal pons and the other variables such as age, gender, disease duration, onset age, BMI, BDI score, MIDAS score or other headache profiles. Migraine diagnostic group (EM vs. CM) was associated with NAA/Cr ratios after controlling for age and sex by the GLM analysis. The estimated differences between EM and CM were 0.203 and 0.151, adjusted *R* ^2^ were 0.143 and 0.098 with *P* values of 0.009 and 0.013 at right and left dorsal pons, respectively. Of note, the results of these calculations were similar with even smaller P values after adding back the outlier data of NAA/Cr in the right dorsal pons.

**Table 2:** Comparisons of the metabolite ratios of ^1^H-MR spectroscopy among controls and patients with episodic and chronic migraine

	Periaqueductal gray				Dorsal pons, right				Dorsal pons, left			
	C	EM	CM	*P*	C	EM	CM	*P*	C	EM	CM	*P*
NAA/Cr ratio mean (SD)	1.45 (0.21)	1.54 (0.19)	1.44 (0.22)	0.228	1.66 (0.38)	1.87 (0.35)	1.64 (0.24)	0.014*	1.58 (0.33)	1.78 (0.26)	1.62 (0.23)	0.034*
Cho/Cr ratio mean (SD)	1.23 (0.26)	1.24 (0.17)	1.20 (0.23)	0.672	1.42 (0.34)	1.44 (0.32)	1.39 (0.21)	0.759	1.37 (0.33)	1.38 (0.28)	1.37 (0.22)	0.994

*NAA* *N*-acetylaspartate, *Cr* creatine, *Cho* choline, *SD* standard deviation, *C* control, *EM* episodic migraine, *CM* chronic migraine

* *P* < 0.05

### Medications overuse (MO)

Patients with CM were further divided into those with (*n* = 30) and without MO (*n* = 23). The demographic data and headache characteristics were compatible between the two subgroups except for earlier age of onset, longer duration of illness and more analgesic usage in the MO subgroup (Table 3). No significant differences in MRS metabolite ratios at the PAG and dorsal pons were noted between these two CM subgroups (Table 4).

**Table 3:** Comparisons of demographics and headache profiles between chronic migraine patients with and without medication overuse

	Without MO (*n* = 23)	With MO (*n* = 30)	*P* value
Age, years	37.8 ± 10.9	42.8 ± 9.7	0.086
Female patients, *n* (%)	18 (78.3)	26 (86.7)	0.419
Age at onset, years	24.4 ± 8.7	19.1 ± 7.5	0.022*
Disease duration, years	14.3 ± 6.2	23.7 ± 10.6	<0.001*
Headache intensity^a^	6.6 ± 2.6	7.4 ± 2.1	0.265
Headache frequency, days/month	24.1 ± 6.4	25.0 ± 5.4	0.586
Analgesics use, days/month	4.4 ± 4.8	25.0 ± 6.3	<0.001*
MIDAS (0–270)	66.9 ± 61.1	59.5 ± 52.7	0.668
BDI score (0–63)	14.8 ± 11.1	14.4 ± 7.8	0.882
BMI (kg/m^2^)	22.3 ± 3.7	22.6 ± 6.4	0.836
Unilateral, *n* (%)	11 (50.0)	16 (53.3)	0.812
Throbbing headache, *n* (%)	14 (63.6)	24 (80.0)	0.189
Physical activity aggravation, *n* (%)	19 (86.4)	21 (70.0)	0.166
Associated symptoms			
Nausea, *n* (%)	13 (59.1)	24 (80.0)	0.100
Vomiting, *n* (%)	10 (45.4)	16 (53.3)	0.575
Photophobia, *n* (%)	17 (77.3)	18 (60.0)	0.240
Phonophobia, *n* (%)	18 (81.8)	21 (70.0)	0.518

*MO* medication overuse, *MIDAS* migraine disability assessment scale, *BDI* Beck depression inventory, *BMI* body mass index

* *P* < 0.05

^a^Headache intensity in a 0–10 numerical rating scale

**Table 4:** Comparisons of the metabolite ratios of ^1^H-MR spectroscopy between chronic migraine patients with and without medication overuse

	Periaqueductal gray			Dorsal pons, right			Dorsal pons, left		
	MO−	MO+	*P*	MO−	MO+	*P*	MO−	MO+	*P*
NAA/Cr ratio mean (SD)	1.48 (0.15)	1.47 (0.27)	0.903	1.63 (0.20)	1.64 (0.30)	0.842	1.59 (0.18)	1.64 (0.29)	0.458
Cho/Cr ratio mean (SD)	1.24 (0.18)	1.23 (0.21)	0.820	1.36 (0.17)	1.38 (0.48)	0.668	1.32 (0.17)	1.38 (0.27)	0.291

*MO* medication overuse, *NAA* *N*-acetylaspartate, *Cr* creatine, *Cho* choline, *SD* standard deviation

## Discussion

Our study showed higher NAA/Cr ratios at bilateral rostral dorsal pons in patients with EM than those in patients with CM and normal controls while the ratios were similar between the latter two groups. Among all migraine patients, the NAA/Cr ratios decreased as the headache frequency or intensity increased, i.e., evolution from EM to CM.

Dorsal pons is the anatomical sites of locus coeruleus and dorsal raphe, the main nuclei of noradrenergic and serotonergic systems. Along with the PAG and other structures, they constitute the descending antinociceptive network which modulates ascending pain signals to the brain [23]. Activation of dorsal pons during migraine attacks has been demonstrated in several studies on patients with EM [9–12]. One MRI study adopting a higher field strength (3 Tesla) showed an increase of gray matter volume at the PAG and dorsal pons in migraine patients with T2-visible hyperintense lesions [13]. Other MRI studies with a lower field strength (1.5 Tesla) showed only cortical but not brainstem gray matter decrease [24]. It is debatable that in morphometric MRI studies, the changes of the gray matter could be attributed to the changes of the cell size (neurons or glia), spine density of neurons or even blood flow or interstitial fluid [24]. In contrast, our MRS study results indicate an involvement of the neurons because of the increment of NAA/Cr levels. It is possible that neurons at the dorsal pons become hypertrophic after repetitive activation in patients with EM.

The exact reason why only EM but not CM patients had higher NAA/Cr levels in the dorsal pons is not clear. If the above theory is correct, the finding suggests that the neuronal hypertrophy might wane during migraine evolution. Therefore, CM might result from decompensation after over-activation of this region, which leads to loss of the hypertrophic response. It is suggested that the brainstem dysfunction may alter cortical and subcortical excitability, which then contributes to migraine evolution [25–28]. Another possibility is that frequent headache attacks might have a disadvantageous effect on neurons at the dorsal pons, leading to neuronal dysfunction or atrophy in patients with CM. In line with the morphometric studies in different pain disorders, chronic pain is related with a decrease of cortical gray matter [15, 24]. Among all migraine patients, our study showed inverse correlations between NAA/Cr ratios and headache frequency or intensity. This may partly explain why we did not demonstrate the difference between normal controls and CM patients. Since this study is of a cross-sectional design, further longitudinal studies are in need to validate this finding. Of note, our study did not find any correlation between NAA/Cr ratio and disease duration, which suggests that EM even for a long duration does not jeopardize such function.

The study results failed to demonstrate the difference of MRS metabolite ratios at the PAG between the two migraine groups and controls. Previous studies have suggested the PAG as an important player in migraine pathogenesis [13, 26]. However, a recent review challenged the role of the PAG in migraine and argued that there is “minimally if any” activation of the PAG in previous neuroimaging studies [16]. We could not completely exclude the possibility of false negativity due to type II error because patients with EM had nonsignificantly higher NAA/Cr level in the PAG compared with the other two groups (Table 2). We also could not exclude the possible confounding effect of the inclusion of the aqueduct (and cerebrospinal fluid) in the VOI of the PAG.

Medication overuse has been recognized as a possible predictor of migraine transformation, but the mechanism has not been determined [2, 29]. Among others, dysfunction of descending antinociceptive network at brainstem and disturbance of serotonin system has been proposed [30]. However, our study failed to show any relationship between MO and brainstem MRS metabolite ratios in patients with CM.

Our study had limitations that are worth noting. First, although we focused on locus coeruleus and dorsal raphe, we could not be certain about the exact relevant substrate within the placed VOIs because the involvement of surrounding structures could not be avoided. Second, patients with EM were younger than the other two groups, though not statistically significant. However, we did not find any correlations between age and NAA/Cr ratios in our participants. Furthermore, the GLM results showed that, after controlling for the age effect, the migraine diagnostic groups (EM vs. CM) remained a significant predictor of NAA/Cr ratios in the dorsal pons. Third, no patients with migraine with aura were included in the EM group; therefore, our study results cannot be generalizable to this patient group. The reason that we recruited only migraine without aura is for comparison’s sake because migraine without aura but not migraine with aura is much more likely to transform to CM according to the ICHD-2 [14].

## References

[1] Eadie MJ (2005). The pathogenesis of migraine—17th to early 20th century understandings. J Clin Neurosci.

[2] Bigal ME, Serrano D, Buse D, Scher A, Stewart WF, Lipton RB (2008). Acute migraine medications and evolution from episodic to chronic migraine: a longitudinal population-based study. Headache.

[3] Committee Headache Classification, Olesen J, Bousser MG, Diener HC, Dodick D, First M, Goadsby PJ, Göbel H, Lainez MJ, Lance JW, Lipton RB, Nappi G, Sakai F, Schoenen J, Silberstein SD, Steiner TJ (2006). New appendix criteria open for a broader concept of chronic migraine. Cephalalgia.

[4] Blumenfeld AM, Varon SF, Wilcox TK, Buse DC, Kawata AK, Manack A, Goadsby PJ, Lipton RB (2011). Disability, HRQoL and resource use among chronic and episodic migraineurs: results from the International Burden of Migraine Study (IBMS). Cephalalgia.

[5] Bigal ME, Serrano D, Reed M, Lipton RB (2008). Chronic migraine in the population: burden, diagnosis, and satisfaction with treatment. Neurology.

[6] Juang KD, Wang SJ, Fuh JL, Lu SR, Su TP (2000). Comorbidity of depressive and anxiety disorders in chronic daily headache and its subtypes. Headache.

[7] Valfre W, Rainero I, Bergui M, Pinessi L (2008). Voxel-Based Morphometry Reveals Gray Matter Abnormalities in Migraine. Headache.

[8] Bigal ME, Lipton RB (2009). What predicts the change from episodic to chronic migraine?. Curr Opin Neurol.

[9] Weiller C, May A, Limmroth V, Jüptner M, Kaube H, Schayck RV, Coenen HH, Diener HC (1995). Brain stem activation in spontaneous human migraine attacks. Nat Med.

[10] Cao Y, Aurora SK, Nagesh V, Patel SC, Welch KM (2002). Functional MRI-BOLD of brainstem structures during visually triggered migraine. Neurology.

[11] Afridi S, Giffin NJ, Kaube H, Friston KJ, Ward NS, Frackowiak RS, Goadsby PJ (2005). A Positron Emission Tomographic Study in Spontaneous Migraine. Arch Neurol.

[12] Stankewitz A, Aderjan D, Eippert F, May A (2011). Trigeminal nociceptive transmission in migraineurs predicts migraine attacks. J Neurosci.

[13] Welch KMA, Nagesh V, Aurora SK, Gelman N (2001). Periaqueductal gray matter dysfunction in migraine: cause or the burden of illness?. Headache.

[14] Rocca MA, Ceccarelli A, Falini A, Colombo B, Tortorella P, Bernasconi L, Comi G, Scotti G, Filippi M (2006). Brain gray matter changes in migraine patients with T2-visible lesions: a 3-T MRI study. Stroke.

[15] May A (2008). Chronic pain may change the structure of the brain. Pain.

[16] Borsook D, Burstein R (2012). The enigma of the dorsolateral pons as a migraine generator. Cephalalgia.

[17] Rudkin TM, Arnold DL (1999). Proton magnetic resonance spectroscopy for the diagnosis and management of cerebral disorders. Arch Neurol.

[18] Wang SJ, Lirng JF, Fuh JL, Chen JJ (2006). Reduction in hypothalamic ^1^H-MRS metabolite ratios in patients with cluster headache. J Neurol Neurosurg Psychiatry.

[19] Safriel Y, Pol-Rodriguez MA, Novotny EJ, Rothman DL, Fulbright RK (2005). Reference values for long echo time MR spectroscopy in healthy adults. AJNR Am J Neuroradiol.

[20] Cox IJ (1996). Development and applications of in vivo clinical magnetic resonance spectroscopy. Prog Biophys Mol Biol.

[21] de Tommaso M, Ceci E, Pica C, Trojano M, Delussi M, Franco G, Livrea P, Ruggieri M (2012). Serum levels of *N*-acetyl-aspartate in migraine and tension-type headache. J Headache Pain.

[22] Headache Classification Committee of the International Headache Society (2004). The International Classification of Headache Disorders, 2nd edn. Cephalalgia.

[23] Heinricher MM, Tavares I, Leith JL, Lumb BM (2009). Descending control of nociception: specificity, recruitment and plasticity. Brain Res Rev.

[24] May A (2009). Morphing voxels: the hype around structural imaging of headache patients. Brain.

[25] Moulton EA, Burstein R, Tully S, Hargreaves R, Becerra L, Borsook D (2008). Interictal dysfunction of a brainstem descending modulatory center in migraine patients. PLoS One.

[26] Aurora SK, Barrodale PM, Tripton RL, Khodavirdi A (2007). Brainstem dysfunction in chronic migraine as evidenced by neurophysiological and positron emission tomography studies. Headache.

[27] Coppola G, Pierelli F, Schoenen J (2007). Is the cerebral cortex hyperexcitable or hyperresponsive in migraine?. Cephalalgia.

[28] Lai KL, Liao KK, Fuh JL, Wang SJ (2011). Subcortical hyperexcitability in migraineurs: a high-frequency oscillation study. Can J Neurol Sci.

[29] Bigal ME, Lipton RB (2008). Excessive acute migraine medication use and migraine progression. Neurology.

[30] Cupini LM, Calabresi P (2005). Medication-overuse headache: pathophysiological insights. J Headache Pain.

